# Supplementary material for: Human Milk Oligosaccharide Profile across Lactation Stages in Israeli Women—A Prospective Observational Study
Source: Nutrients. 2023 May 30;15(11):2548. doi: 10.3390/nu15112548 (PMC10255315; doi:10.3390/nu15112548)
Supplement: Supplementary file 1 [file nutrients-15-02548-s001.zip › nutrients-2322218-supplementary.pdf]

Table S1. Summary of retention times, and accurate masses of the parent and daughter ions used for identification and quantification of the nine HMOs (the MS/MS spectra of individual HOMs).

Analyte Name	RT, min	Atomic Composition	Origin of the parent ion	Accurate mass of the parent ion	Distinctive MS/MS fragment ions
2-Fucosyllactose (2-FL)	8.15	$C_{18}H_{32}O_{15}$	$[M+HCOO]^-$	533.1723	409.1346, 205.0713
3-Fucosyllactose (3-FL)	8.29	$C_{18}H_{32}O_{15}$	$[M+HCOO]^-$	533.1723	179.0559
3’-Sialyllactose (3’-SL)	9.23	$C_{23}H_{39}NO_{19}$	$[M-H]^-$	632.2044	290.0879
6’-Sialyllactose (6’-SL)	9.53	$C_{23}H_{39}NO_{19}$	$[M-H]^-$	632.2044	290.0879, 470.1511, 572.1825
Disialyl-lacto-N-tetralose (DSLNT)	11.18	$C_{48}H_{79}N_3O_{37}$	$[M-2H]^{2-}$	643.7123	290.0877, 997.3387
Lacto-N-tetraose (LNT)	9.14	$C_{26}H_{45}NO_{21}$	$[M-H]^-$	706.2411	142.0506, 202.0717
Lacto-N-fucopentaose I (LNFP I)	9.64	$C_{32}H_{55}NO_{25}$	$[M+HCOO]^-$	898.3045	205.0713, 325.1137
Lacto-N-fucopentaose II (LNFP II)	9.75	$C_{32}H_{55}NO_{25}$	$[M+HCOO]^-$	898.3045	288.1086, 348.1298
Lacto-N-fucopentaose III (LNFP III)	9.75	$C_{32}H_{55}NO_{25}$	$[M+HCOO]^-$	898.3045	179.0559, 364.1244

Table S2: Absolute concentrations (nmol/mL) of individual HMO in all available HM samples

	All Secretors samples	All Non-secretors samples	All HM samples
HMO	n=30	n=23	n=53
2'FL	1788 ± 223.2	0	1012 ± 175.6
3FL	182.9 ± 23.57	414.1 ± 57.84	283.3 ± 32.28
3'SL	97.17 ± 10.51	81.03 ± 8.621	90.17 ± 7.054
6'SL	192.2 ± 20.16	190.8 ± 25.06	191.6 ± 15.61
DSLNT	158.1 ± 14.01	191.2 ± 19.78	172.4 ± 11.79
LNFP I	819.4 ± 231.3	0	463.8 ± 141.6
LNFPII	85.56 ± 23.29	311.8 ± 88.59	183.7 ± 43.06
LNFPIII	63.49 ± 11.34	156.3 ± 34.79	103.8 ± 17.42
LNT	354.2 ± 42.39	630.6 ± 122.1	474.1 ± 60.54

Data are expressed as mean ± SEM

Figure S1:

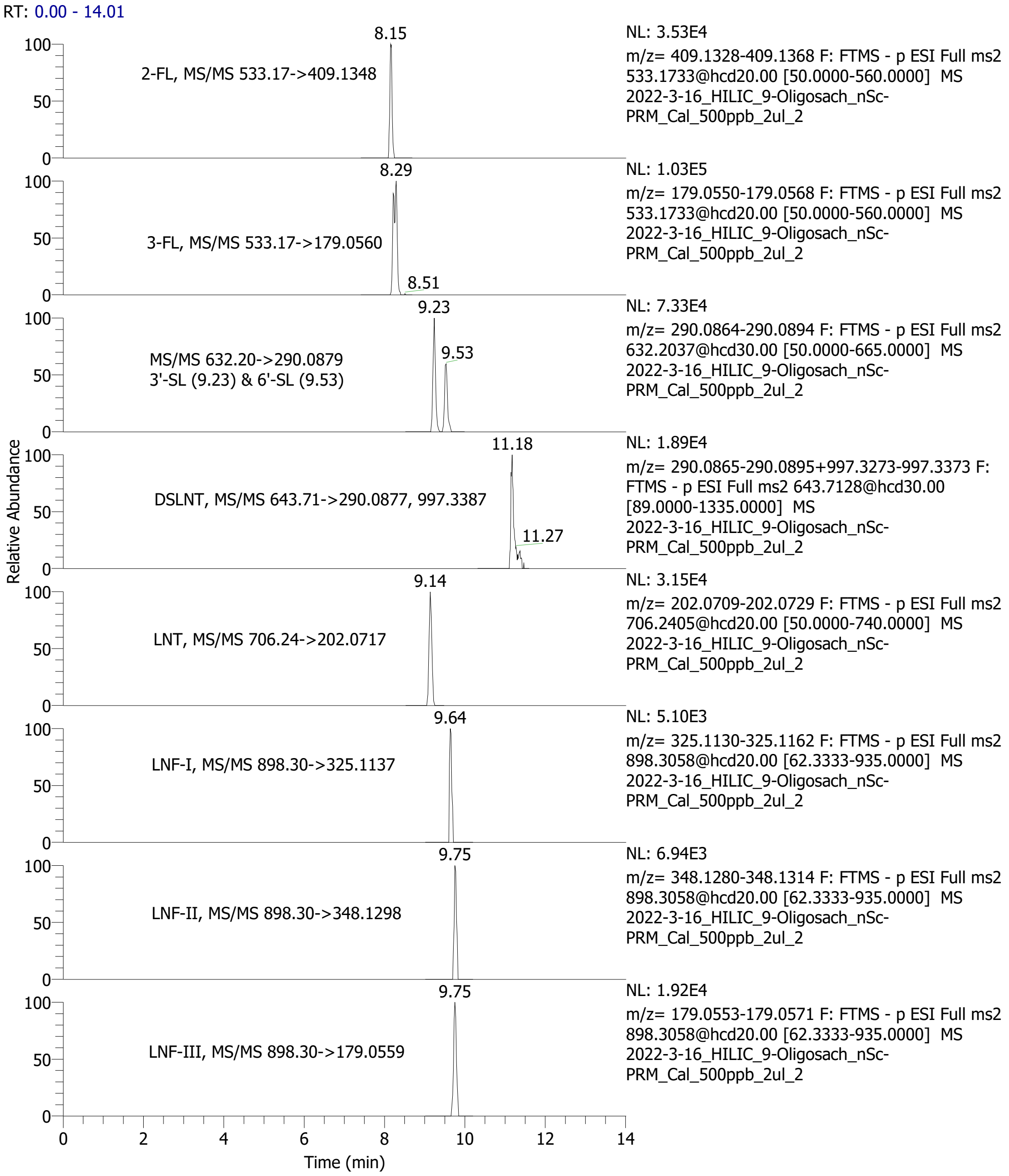

Figure S1: Negative ESI LC-MS/MS (PRM) extracted accurate mass ion chromatograms of nine analyzed HMOs. Co-eluted isomeric HOMs could be identified and distinguished by their characteristic MS/MS spectra.

Figure S2

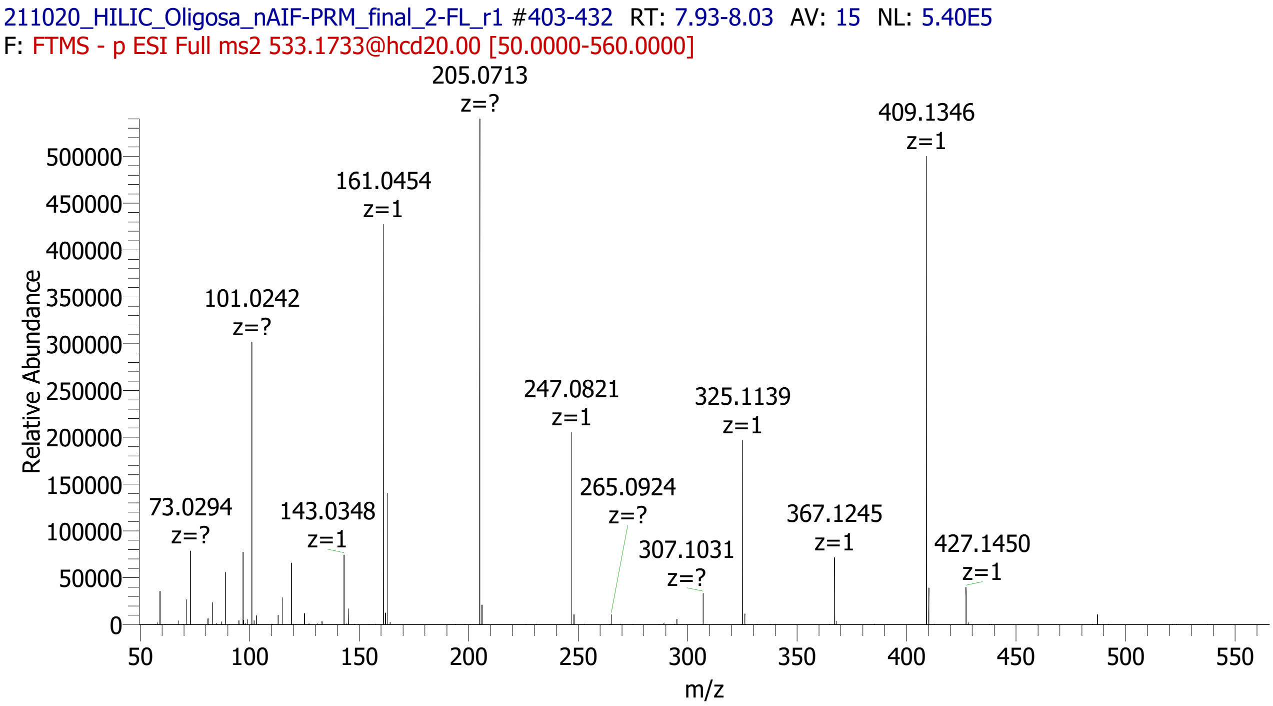

Figure S2A: Negative ESI CID mass spectrum of 2-FL [M+HCOO]⁻ ion.

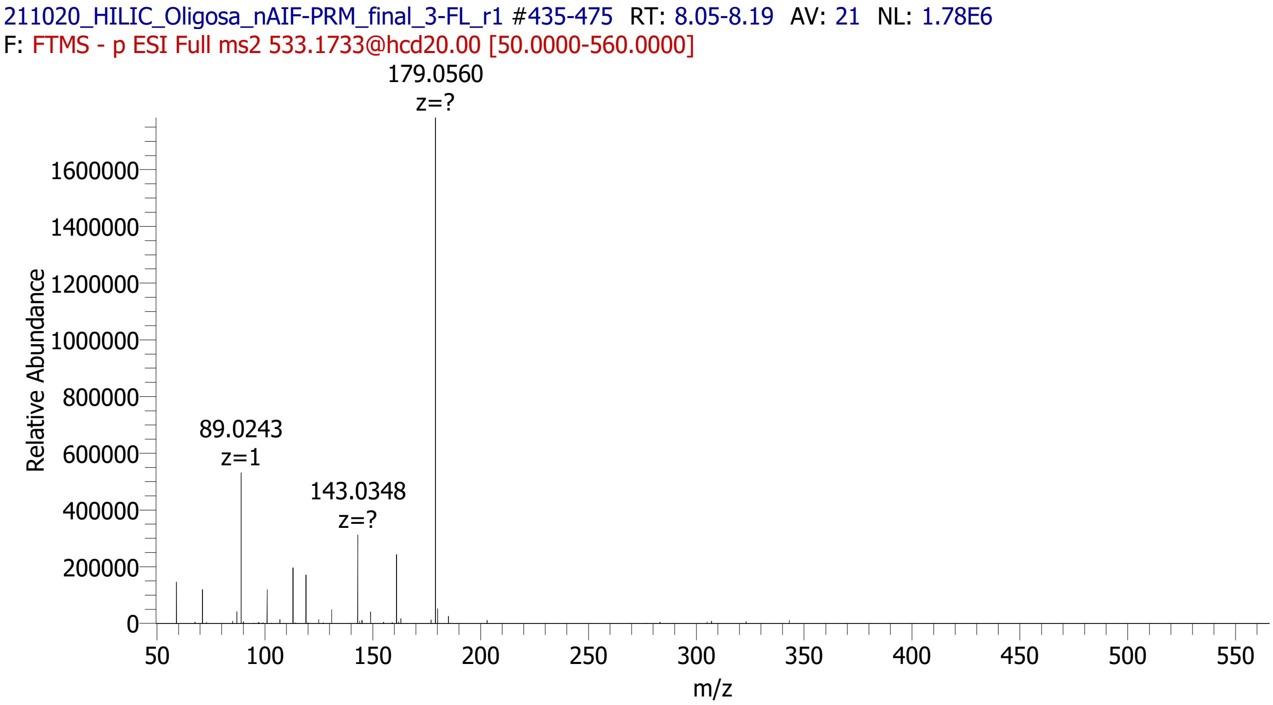

Figure S2B: Negative ESI CID mass spectrum of 3-FL [M+HCOO]⁻

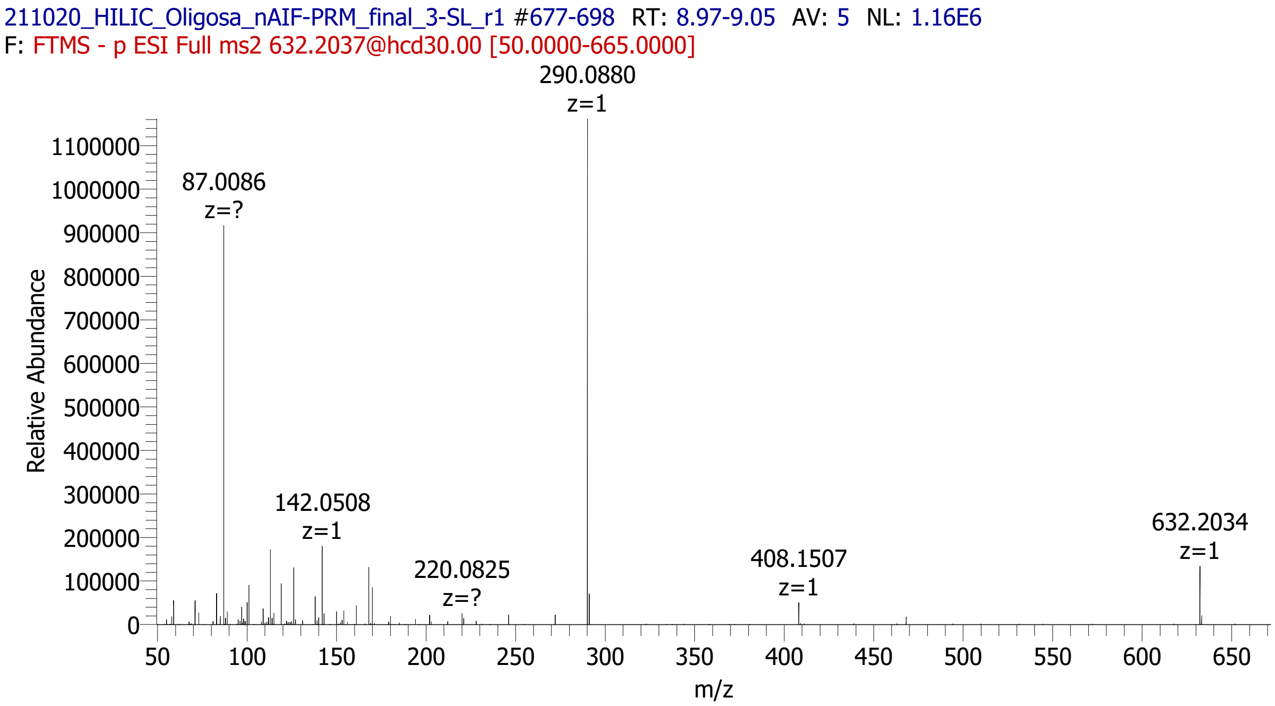

Figure S2C: Negative ESI CID mass spectrum of 3’-SL [M-H]⁻ ion.

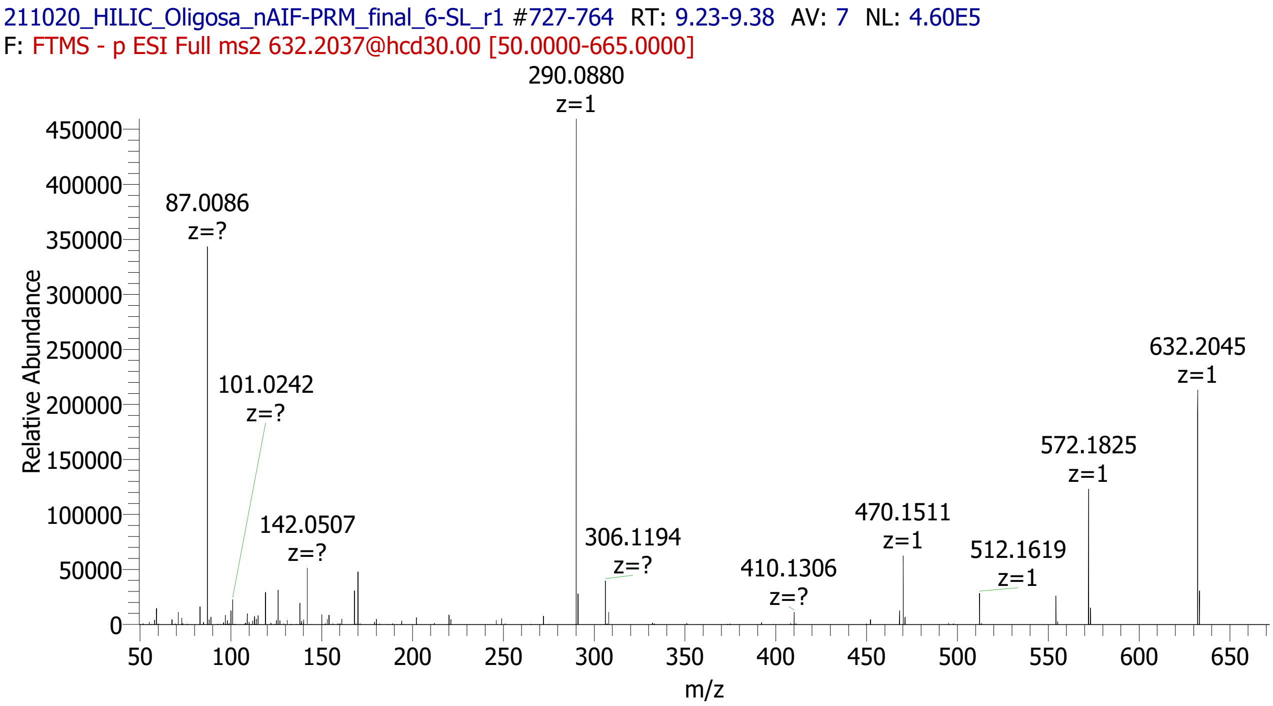

Figure S2D: Negative ESI CID mass spectrum of 6’-SL [M-H]⁻ ion.

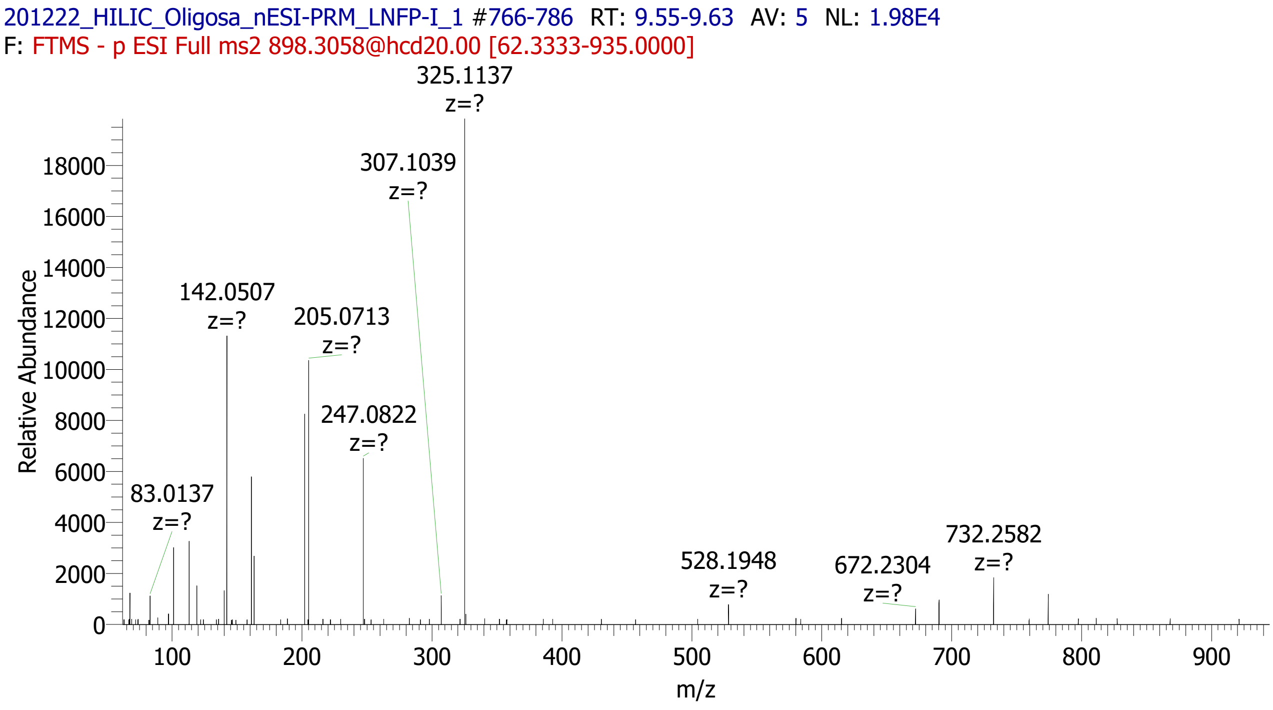

Figure S2E: Negative ESI CID mass spectrum of LNF I [M+HCOO]$^-$ ion.

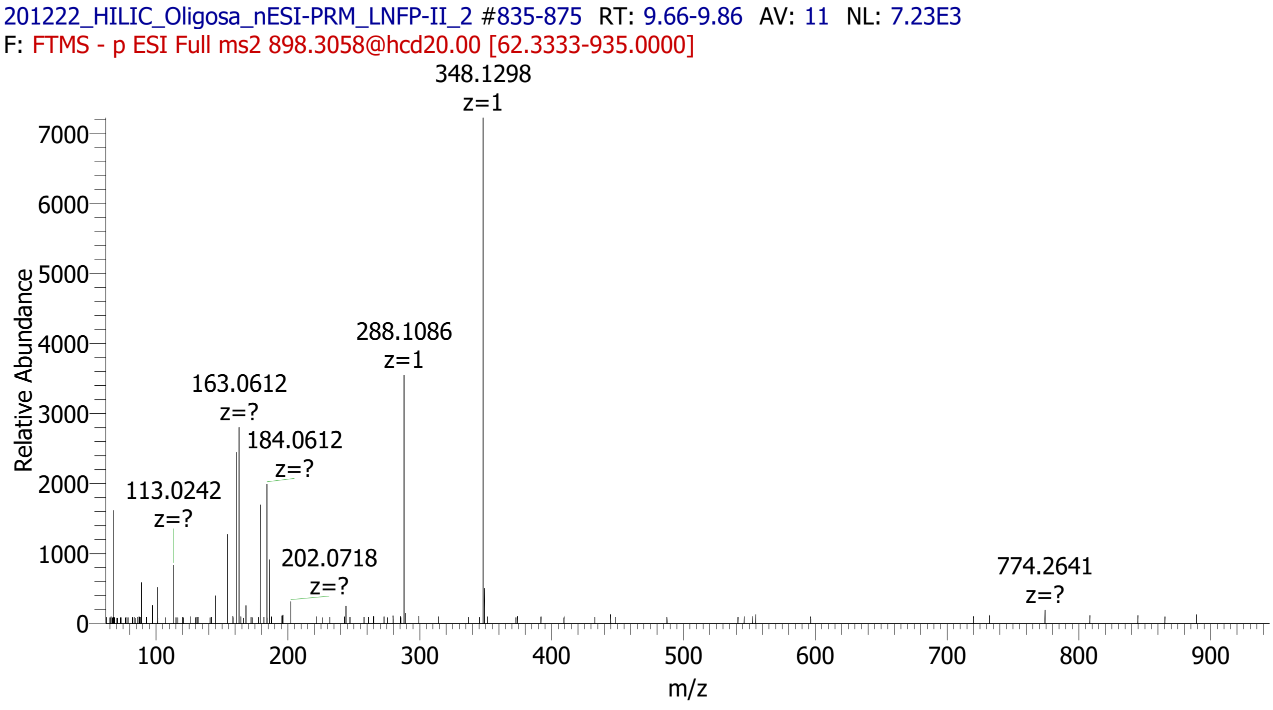

Figure S2F: Negative ESI CID mass spectrum of LNF II [M+HCOO]$^-$ ion.

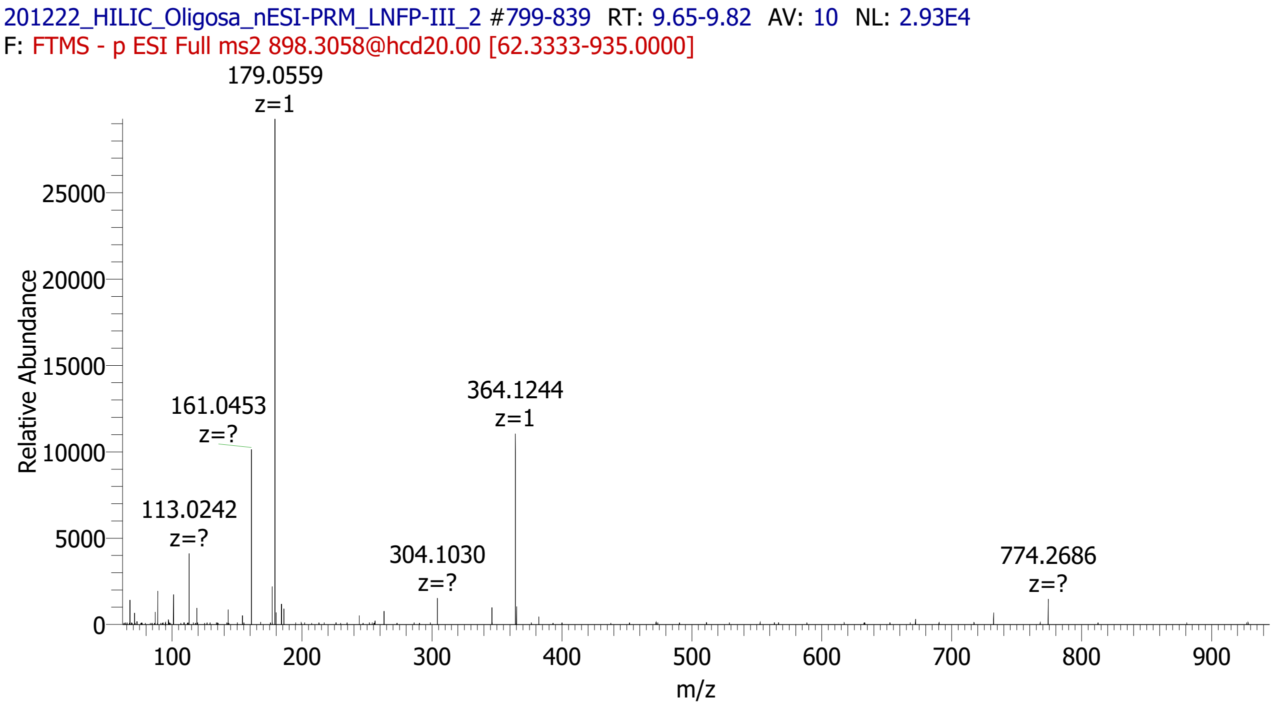

Figure S2G: Negative ESI CID mass spectrum of LNF III [M+HCOO]$^{-}$ ion.

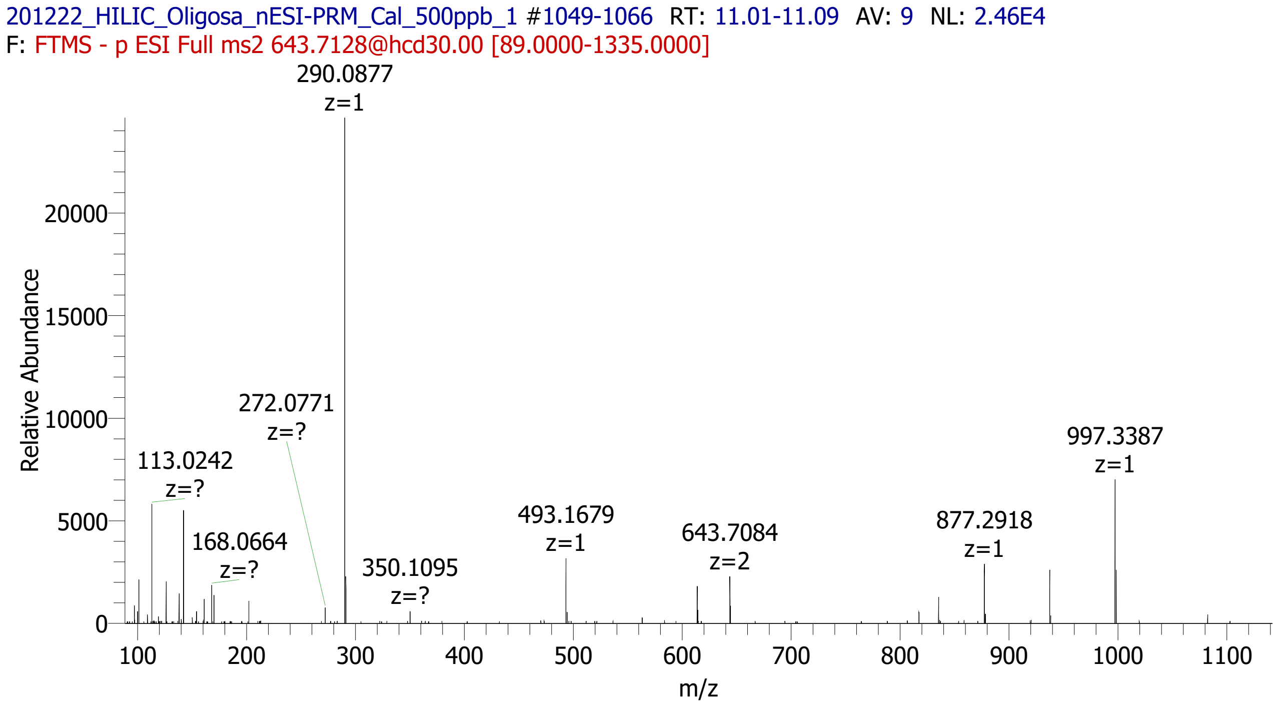

Figure S2H: Negative ESI CID mass spectrum of DSLNT [M-2H]$^{2-}$ ion.

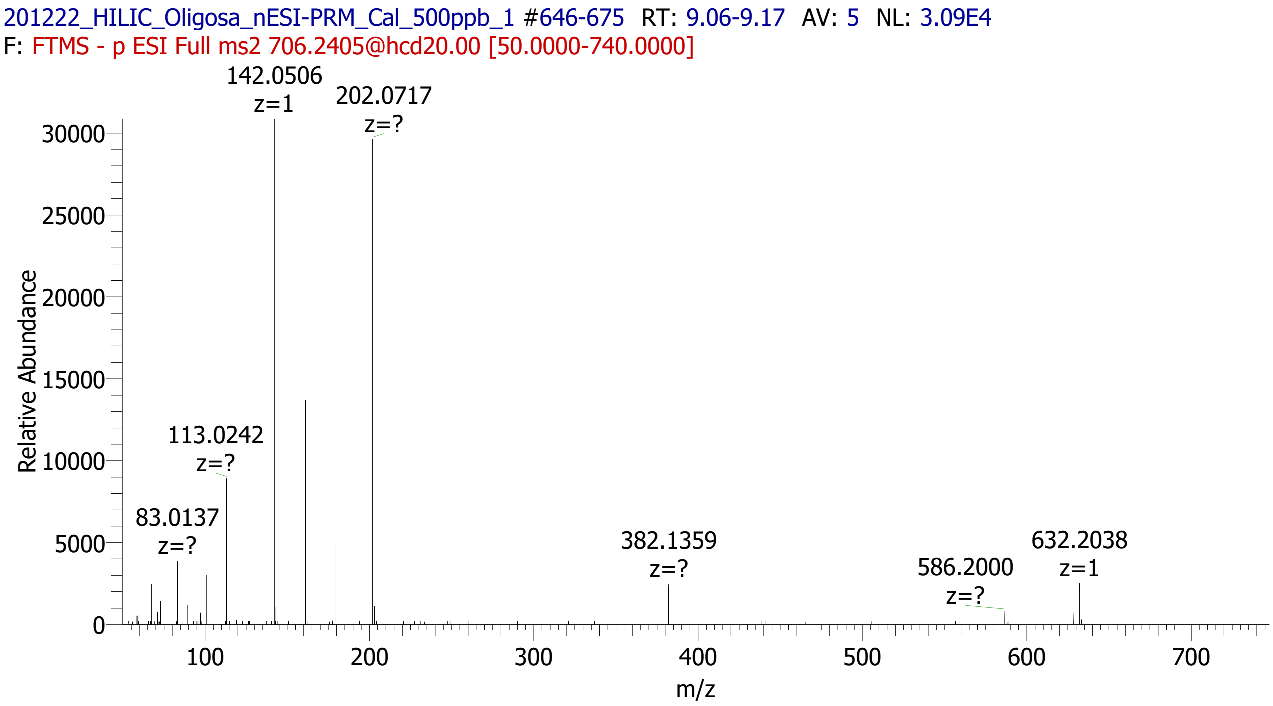

Figure S2I: Negative ESI CID mass spectrum of LNT [M-H]⁻ ion.
